# High altitude area – A risk factor for gastric perforation? : A case series

**DOI:** 10.1016/j.ijscr.2018.11.037

**Published:** 2018-11-22

**Authors:** Aditya Pawar, Vikas Sinha, Pawan Sharma, Deependra Kumar Sinha

**Affiliations:** a Department of Surgery, Military Field Hospital, Pratappur, Leh, J & K, India; b Department of Anaesthesiology, Military Field Hospital, Pratappur, Leh, J & K, India; c Department of Surgery, Command Hospital, Udhampur, J & K, India; d Department of Anaesthesia and Intensive Care, Command Hospital, Udhampur, J & K, India

**Keywords:** HAA, high altitude area, PUD, peptic ulcer disease, PPU, perforated peptic ulcer, LMICs, low & middle income countries, NSAIDs, non steroidal anti- inflammatory drugs, Hb, haemoglobin, TLC, total leucocyte count, DLC, differential leucocyte count, High altitude, Gastric antrum, Perforations, Mucosal atrophy, Intra-luminal pressure

## Abstract

**Introduction:**

This is an original case series on soldiers evacuated from HAA(High Altitude Area) over last one year(Sept 2017–Aug 2018), of which 100% cases were found to have gastric antrum perforation. This is a deviation from the usual trend that is being noted in Indian subcontinent where duodenal perforations are commonest.

**Discussion:**

High altitude associated dyspepsia is a common phenomenon and some studies done in high altitude population have recorded high incidence of antral gastritis and mucosal atrophy on histo-pathological evaluation. This is also supported by high incidence of *H. pylori* infection. The presence of atrophic gastric mucosa associated with antral gastritis, when exposed to hypobaric hypoxemic conditions faced by soldiers posted to heights above 15,000 ft, may leads to increased intra-luminal pressure and ischemia which probably predisposes individuals for gastric antrum perforations.

**Conclusion:**

This is a relatively untouched topic as we have not found studies on*high altitude related gastric perforations* and probably this is the first case series of its kind. The acknowledgement of this phenomenon may pave way for further studies for defining the role of high altitude in gastric perforations.

## Highlights

• Original study.
• Based on an interesting variation in location of perforated peptic ulcers observed in soldiers posted to high altitude areas.
• Literature review and research suggestive of possibility of high altitude induced patho-physiological changes behind gastric perforations.
• This study is probably first of it’s kind and may be pioneering for encouraging further studies to establish a definite role of high altitude in causing gastric perforations.

## 1 Introduction

This is an original work. It has been created in compliance with SCARE and PROCESS guidelines [1,2]. This is a retrospective observational study.

Perforated peptic ulcers is considered amongst the most feared surgical emergency globally with short term mortality rates reaching up to 30% [3]. There are various risk factors identified for PUD(Peptic Ulcer Disease) but the exact mechanism for PPU(Perforated Peptic Ulcer) is still not clear.

PPU have seen a constant variation in epidemiology since last century, where the disease has noted a decreasing incidence after 1950 in men, however in women the incidence has seen a slight up-trend after 1950 [4,5].

The age group affected in various western literature has also seen a shift in trend from a disease of young initially to that of middle aged and elderly [4,6].

Data from Low and Middle income countries (LMIC’s), suggest that the median age at diagnosis of PPU has seen an increase of two decades i.e. from 30 to 40 s earlier to 60 s and above presently [7].

The variation has been attributed primarily to socio-economic development, prevalence of Helicobacter pylori infection, smoking habits, use of NSAIDS & eating habits [[8], [9], [10], [11], [12], [13]].

In certain studies the perforation frequency has been related to the geographical distribution of *H. pylori*, where more duodenal perforations have been reported in areas with higher prevalence of *H. pylori* infection [14].

Compared to the western countries where the rates of duodenal perforations have fallen steadily and that of gastric perforation have remained more or less stable, the data from LMICs suggest high rates of duodenal perforation as compared to gastric perforation with certain studies showing duodenal perforation in 90% cases [15,16].

Studies done in Indian subcontinent are also suggestive of predominant duodenal ulcer perforation with one study showing a ratio of 12.7 : 1 for duodenal to gastric perforation [17,18].

Interestingly we here at a forward Military Field Hospital located at an altitude of 10,500 ft have noted a trend which does not corroborates with the existing trend in rest of the country. We provide medical and surgical cover to troops stationed in Siachen Glacier (Himalayan range) which happens to be the world’s highest battle field where the average altitude of post is 15,000 ft and the average duration of stay is 60–90 days.

We have received young soldiers (<30 yrs) with perforation peritonitis who have been detected with gastric antrum perforation on exploratory laparotomy. This predisposition of gastric antrum perforation in young soldiers defy the usual trend of this geographical location, prompting us to believe that this variation from the usual could be due to patho-physiological changes induced by prolonged stay in HAA(High Altitude Area)

## 2 Case presentation

**Fig. 1:**
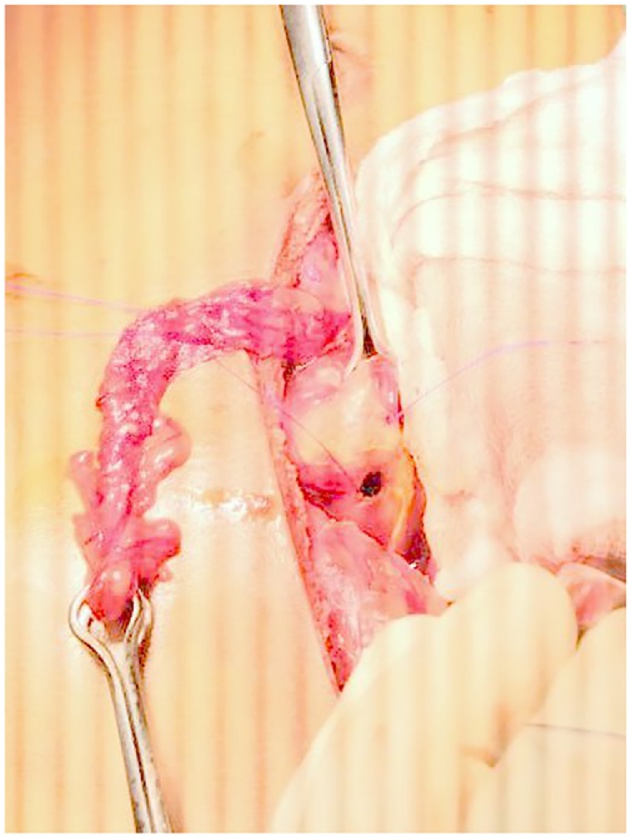
1 × 1 cm gastric antrum perforation with a tailored omental tongue.
2 CASE TWO (Aug-2018)-: A 27 yrs old soldier, non smoker not on NSAIDS and not a known case of PUD, was on long range patrolling at an altitude of 17,000 ft, when he developed sudden onset pain which was initially at epigastric region then progressed to involve rest of abdomen and was associated with one episode of vomiting. Patient was evacuated by road and reached the hospital after 10 h. At presentation patient had tachycardia of 110 bts/min and B.P of 112/70 mmHg. Per abdomen revealed diffuse tenderness with rigidity. Investigations- Xray chest PA view s/o- gas under diaphragm. Hb- 15.4gm%, TLC- 7000 cells/mm3, DLC- N-87,L-10,M-01,E-02. He underwent emergency exploratory laparotomy using a supra-umblical incision. A 0.5 x 0.5 cm punched out gastric antrum perforation was found. Surrounding tissue found to be normal with only features of inflammation. Modified Graham’s Omental Patch Repair was done. Post-op recovery uneventful (Fig. 2).

1 CASE ONE (Aug-2018)-: A 26 yrs old soldier, resident of Maharashtra(plains), India. A non smoker, not on NSAIDS and not a known case of PUD, after completion of 120 days of tenure at 18,000 ft, while de-inducting developed pain abdomen in epigastric region followed by diffuse pain. Patient evacuated by helicopter and on presentation was found to have tachycardia and dehydration. Per abdominal findings revealed severe tenderness and rigidity. Investigations- Xray chest PA view s/o- gas under diaphragm. Hb- 21gm%, TLC- 7700 cells/mm3 DLC- N-82, L-11, M03, E-04 Patient was introduced with epidural catheter for intra-op & post-op pain management. He underwent emergency exploratory laparotomy using a supra-umblical incision. A 1 × 1 cm punched out gastric antrum perforation was found. Surrounding tissue found to be normal with only features of inflammation. Modified Graham’s Omental Patch Repair was done. Post-op recovery uneventful (Fig. 1).

**Fig. 2:**
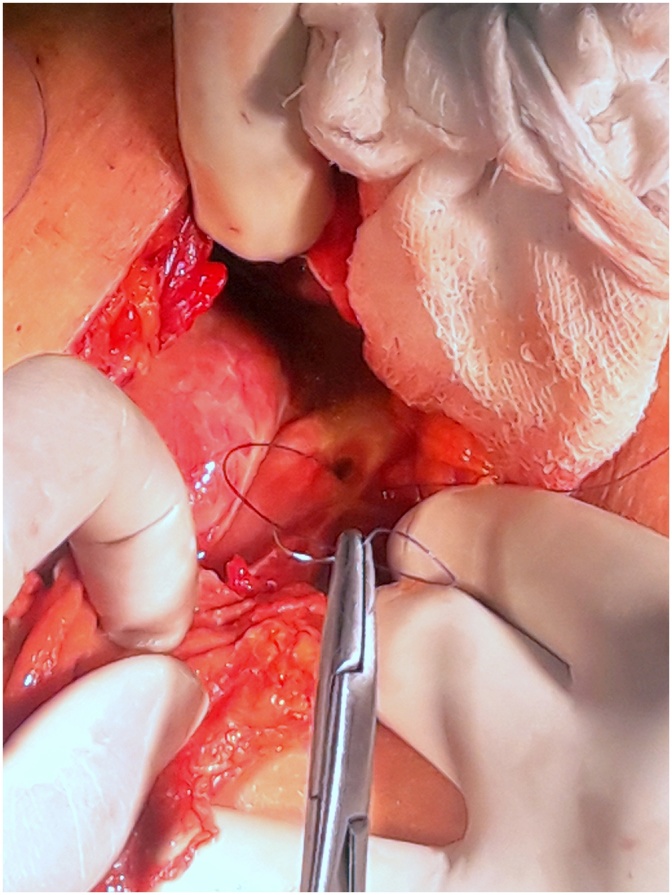
0.5 × 0.5 cm gastric antrum perforation with stay suture in place.
3 CASE THREE(Oct-2017)-: A 24 yrs old soldier, non smoker not on NSAIDS and not a known case of PUD, was evacuated from a post located at 19,263 ft with complaints of diffuse pain abdomen which started from the epigastric region. Per abdomen revealed diffuse tenderness with rigidity. Investigations- Xray chest PA view s/o- gas under diaphragm. Hb- 18.8gm%, TLC- 15,400 cells/mm3, DLC- N-72,L-23,M-02,E-03. He underwent emergency exploratory laparotomy using a supra-umblical incision. A punched out gastric antrum perforation was found. Surrounding tissue found to be normal with only features of inflammation. Modified Graham’s Omental Patch Repair was done. Post-op recovery uneventful (Fig. 3).

**Fig. 3:**
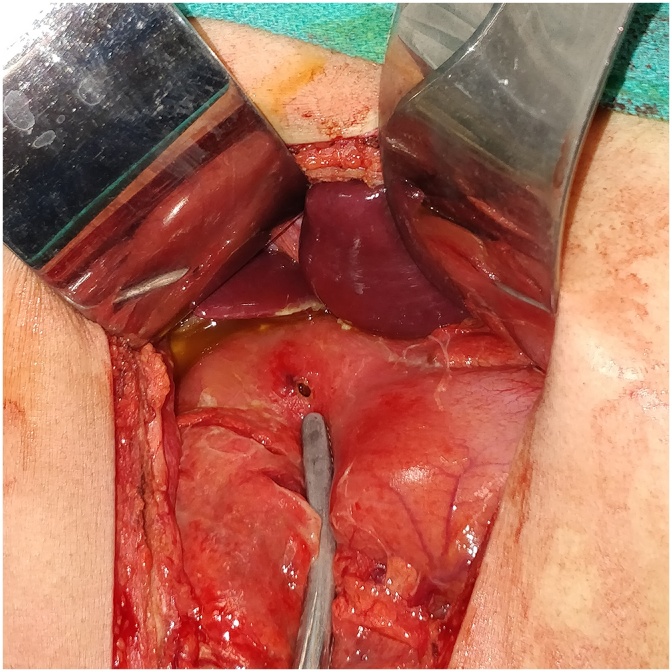
Gastric Antrum Perforation.
4 CASE 4 (Oct-2017)-: A 36 yrs old soldier, non smoker not on NSAIDS and not a known case of PUD, was evacuated from a post located at 19,460 ft with complaints of sudden onset pain involving the epigastric region which became diffuse involving the whole abdomen. Per abdomen revealed tenderness with diffuse guarding and rigidity. Investigations- Xray chest PA view s/o- gas under diaphragm. Hb- 22.3gm%, TLC- 14,500 cells/mm3, DLC- N-84,L-11,M-02,E-03. Epidural catheter was introduced for intra-op and post-op pain management. He underwent emergency exploratory laparotomy using a midline incision, extending from xiphi-sternum to infra-umbilical region. A punched out gastric antrum perforation was found. Surrounding tissue found to be normal with only features of inflammation. Primary repair was done. Post-op recovery uneventful (Fig. 4).

**Fig. 4:**
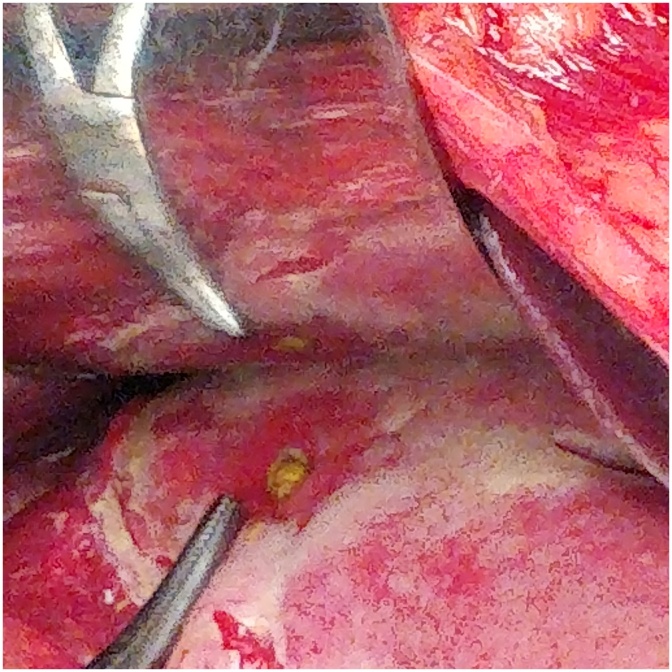
Gastric Antrum Perforation.
5 CASE 5 (Sept-2017)-: A 28 yrs old male soldier, smoker for last 17 yrs. Not on NSAIDS and not a known case of PUD, was evacuated from a post located at 11,000 ft with complaints of sudden onset pain involving the epigastric region which became diffuse involving the whole abdomen. Per abdomen revealed severe tenderness with rigidity. Investigations- Xray chest PA view s/o- gas under diaphragm. Hb- 17.6gm%, TLC- 14,100 cells/mm3, DLC- N-82,L-14,M-01,E-03. Epidural catheter was introduced for intra-op and post-op pain management. He underwent emergency exploratory laparotomy using a midline incision, extending from xiphi-sternum to infra-umbilical region. A punched out gastric perforation was found in antro-pyloric region. Surrounding tissue found to be normal with only features of inflammation. Primary repair was done. Post-op recovery uneventful (Fig. 5).

**Fig. 5:**
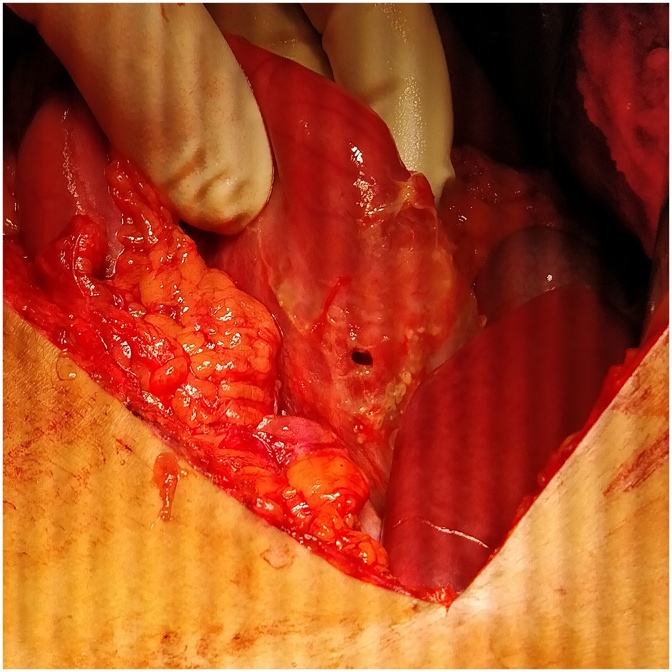
Gastric Antrum Perforation.

## 3 Discussion

This forward military field hospital located at High Altitude, providing forward medical & surgical care to troops located at an altitude >15,000 ft provide us with a rare opportunity to witness High Altitude illnesses and injuries.

The surgical emergencies in the form of acute abdomen due to hollow viscus perforation that we have managed from Sept 2017–Aug 2018, have all been found to be distal stomach perforations. The age group affected is young with all individuals below 30 years of age barring one case.

This trend of young individuals who are fit soldiers with no previous history of smoking, NSAIDs intake or previous history of PUD, defies the usual etio-pathological and geographical trends noted in this part of the world.

This 100% involvement of the distal stomach in cases evacuated from High Altitude Area without any obvious risk factors except H.pylori infections have surprised us as clinicians.

Also a seasonal variation with all cases being reported in the months of Jul-Oct was noted.

Dyspepsia at high altitude is a noted fact. A study done on local population of Leh & Ladhak(J & K, India) suggested predominant finding of antral gastritis in 71% of the studied population. H.pylori was documented in 93% of cases and histopathology revealed mild to moderate atrophy [19]. Another study compared histopathology findings in H.pylori patients living in high altitude area with patients living at sea level, found antrum being the most commonly affected location with atrophic gastritis and total deep gland loss as the most common finding with mountain population being more severly affected [20].

The data on increase in intra-luminal pressure leading to hollow viscus perforation is sparse and the first such reported case is from a case report of a colonic perforation during a high altitude flight where the authors have explained the perforation based on expansion of gas in accordance with Boyle’s law and La Place’s law [21].

Thus in the backdrop of severe antral gastritis and H pylori infection seen in population living at high altitude the possibility of these factors predisposing to gastric antrum perforation in presence of hypobaric hypoxemic conditions leading to raised intra-luminal pressure and ischemia, could provide a possible explanation to the gastric perforations noted by us.

## 4 Conclusion

An unusual observation of perforations located at gastric antrum in young soldiers posted at high altitude area (>15,000 ft) defies the usual epidemiological trends of PPUs. There has been a surprising 100% involvement of gastric antrum in cases evacuated to this forward high altitude military facility over last one year. This observation in the backdrop of high incidence of antral gastritis and H.pylori infection seen in high altitude based population and prolonged exposure to hypobaric hypoxemic conditions faced by soldiers posted to high altitude areas may have a role in pathogenesis of events that have lead to predisposition of gastric antrum to perforate. Our proposition is also supported by the fact that intra-luminal pressure increases as individual is exposed to altitude above sea level in accordance with Boyle’s & Laplace’s law.

Hence we would like to conclude that probably prolonged exposure to high altitude induces patho-physiological changes that makes predisposed individuals to develop gastric antrum perforations. However before High Altitude is attributed as a risk factor for gastric perforations further detailed studies are suggested in order to provide more insight into etio-pathogenesis of gastric perforations at High Altitude Area so that relevant measures, screening protocols and prophylaxis can be implemented to prevent fatal emergencies as these.

## Conflicts of interest

No Conflict of Interest.

## Funding

No sources of funding involved.

## Ethical Approval

Ethical Clearance not required. A clearance from within the organisation was taken as this is an observational retrospective study where no variation from the institutional standards in terms of investigations, treatment and any other component of overall management was made.

## Consent

Written informed consent obtained.

## Author contribution

1 Major (Dr) Aditya Pawar (First Author)-: The author has been involved with the conceptualisation, researching, literature review, data collection and drafting of the manuscript. He was the operating surgeon in Case 1 &2 of the case series.
2 Major (Dr) Vikas Sinha (Second Author)-: The author has been involved with the conceptualisation, researching and data collection. He was the Anaesthesiologist in all the cases except Case 2. He has also been the source of intra-op images provided in this case series.
3 Col (Dr) Pawan Sharma (Third Author)-: The author has been involved in proof reading, editing and overall guidance.
4 Col (Dr) Deependra Kumar Sinha (Fourth Author)-: The author has been involved in proof reading, editing and overall guidance

## Registration of Research Studies

The study has been registered with ClinicalTrials.gov and the ClinicalTrial.gov Identifier Number (UIN) : NCT03724513.

## Guarantor

Maj. (Dr).Aditya Pawar.
